# Contribution of *MTHFR* C677T and A1298C Variants to Retinoblastoma Risk in Mexican Children

**DOI:** 10.3390/genes17101204

**Published:** 2026-09-29

**Authors:** Jose de Jesus Perez-Becerra, Juan Antonio Ramirez-Corona, David Fernandez Sanchez, Fernando Alexis Flores Leura, Sinhue Alejandro Brukman-Jimenez, Alfredo Corona-Rivera, Jorge Román Corona-Rivera, Graciela Gonzalez-Perez, Gladys Hassel Calderon-Camacho, Andrea Montserrat Figueroa Pizano, Janette Alicia Mena Leon, Mireya Orozco-Vela, Lucina Bobadilla-Morales

**Affiliations:** 1 Human Genetics PhD Program, Department of Molecular Biology and Genomics, Centro Universitario de Ciencias de la Salud, Universidad de Guadalajara, Guadalajara 44340, Mexico; perezbecerra95@gmail.com (J.d.J.P.-B.);; 2 Human Genetics Institute “Dr. Enrique Corona Rivera”, Department of Molecular Biology and Genomics, Centro Universitario de Ciencias de la Salud, Universidad de Guadalajara, Guadalajara 44340, Mexico; 3 Cytogenetics Unit, Hospital Civil de Guadalajara Dr. Juan I. Menchaca, Guadalajara 44340, Mexico; 4 Center for Registry and Research in Congenital Anomalies (CRIAC), Service of Genetics and Cytogenetics Unit, Pediatrics Division, Dr. Juan I. Menchaca Civil Hospital of Guadalajara, Guadalajara 44340, Mexico; 5 Ophthalmology Department, Hospital Civil de Guadalajara Fray Antonio Alcalde, Guadalajara 44280, Mexico; 6 University Center of Health Sciences, University of Guadalajara, Guadalajara 44360, Mexico

**Keywords:** folate, *MTHFR*, polymorphism, retinoblastoma

## Abstract

**Background**: Retinoblastoma (RB) is the most common malignant intraocular tumor in childhood. Although germline and somatic alterations in RB1 contribute to RB development, additional genetic factors may influence susceptibility. Methylenetetrahydrofolate reductase (MTHFR) participates in folate metabolism and DNA synthesis and repair. This study evaluated the association of *MTHFR* C677T and A1298C polymorphisms with RB susceptibility in Mexican children. **Methods**: A case–control study included 58 Mexican children with clinically diagnosed RB and 301 healthy controls matched for sex and ethnicity. Genomic DNA was extracted from peripheral blood, and *MTHFR* C677T (rs1801133) and A1298C (rs1801131) were genotyped using TaqMan allelic discrimination assays. Allele and genotype frequencies were compared using chi-square and Fisher’s exact tests. Odds ratios (ORs) and 95% confidence intervals (CIs) were calculated. A recessive genetic model was applied to both polymorphisms. **Results**: The *MTHFR* 677TT genotype was associated with significantly reduced odds of RB under the recessive genetic model (TT vs. CC + CT), using Firth’s penalized logistic regression (OR = 0.066; 95% CI: 0.007–0.250, *p* < 0.001). The T allele was also less frequent among cases, supporting a potential protective association. No significant association was observed between *MTHFR* A1298C and RB susceptibility (*p* = 0.262). **Conclusions**: The *MTHFR* C677T polymorphism was associated with RB susceptibility in this Mexican cohort, with the TT genotype showing a potential protective association. No significant association was identified for A1298C. Further studies are warranted to investigate the role of MTHFR variation and folate-dependent DNA metabolism in RB susceptibility.

## 1. Introduction

Retinoblastoma (RB) is the most common malignant intraocular tumor in childhood, accounting for up to 4% of all pediatric cancers, with an estimated global incidence of one in 15,000 to 20,000 live births [1]. Although survival rates can reach up to 95% in countries with early access to medical care, the clinical burden is significantly higher in low-income countries, where delayed diagnosis often leads to poor outcomes [2].

Approximately 40% of RB cases are known to have a hereditary component, primarily associated with germline mutations in the *RB1* gene. However, most cases arise sporadically due to somatic mutations in cone precursor cells [3]. Beyond *RB1* mutations, susceptibility to RB may be influenced by a combination of environmental exposures and other genetic risk factors [4,5]. Among these, variants in genes that participate in the folate–methionine pathway, which is involved in DNA synthesis, repair, and epigenetic regulation through methylation, have gained increasing attention.

The folate–methionine pathway plays a key role in several essential cellular processes, including purine and pyrimidine synthesis, histone and DNA methylation, and maintenance of cell cycle homeostasis. Disruptions in this pathway may impair genomic stability and alter methylation patterns, potentially promoting tumor initiation and progression. The enzyme methylenetetrahydrofolate reductase (MTHFR) mediates the formation of 5-methyltetrahydrofolate from 5,10-methylenetetrahydrofolate. This product subsequently acts as a cofactor in the remethylation of homocysteine, leading to methionine synthesis. Methionine is subsequently converted to S-adenosylmethionine, the principal methyl donor in gene methylation processes [6].

Two common and extensively studied polymorphisms in the *MTHFR* gene are C677T (rs1801133) and A1298C (rs1801131). The T allele of C677T results in a missense variant (Ala222Val) that reduces the enzyme’s thermal stability and decreases its enzymatic activity by up to 70% in individuals homozygous for the T allele (TT genotype). In contrast, the A1298C variant leads to a Glu429Ala substitution and is associated with a moderate reduction in enzymatic function [7]. These variants may lead to elevated plasma homocysteine levels and abnormal methylation patterns, which have been implicated in various diseases, including neural tube defects, cardiovascular disorders, and several types of cancer [8,9].

Recent studies have investigated the association between *MTHFR* polymorphisms and the risk of RB [10,11,12,13]. A recent meta-analysis that pooled data from multiple studies conducted in Asia and South America identified a significant association between the C677T variant and RB risk. However, no association was observed for the A1298C variant [14].

In Mexico, the frequency of *MTHFR* polymorphisms is relatively high in the general population. It is estimated that more than 50% of Mexicans carry the T allele at the C677T locus [15]. This high allele frequency is of particular clinical relevance, not only for folate-related metabolic disorders, but also for malignancies in which epigenetic dysregulation and altered nucleotide metabolism are involved. Nevertheless, the potential association between *MTHFR* C677T and A1298C variants and the risk of RB in Mexican children has not yet been explored.

Therefore, this study aims to evaluate the association between the *MTHFR* C677T and A1298C polymorphisms and the risk of developing RB in a cohort of Mexican children. Specifically, we seek to determine whether certain alleles or genotypes confer increased susceptibility or a protective effect against tumor development. Findings from this study may contribute to a better understanding of genetic risk factors for RB and support the identification of potential molecular markers for early detection or therapeutic intervention.

## 2. Materials and Methods

**Study Population**

This case–control study included 58 Mexican children with a clinical diagnosis of retinoblastoma (RB) who were recruited from the Hospital Civil de Guadalajara “Dr. Juan I. Menchaca” as the case group.

The control group consisted of 301 healthy live-born infants enrolled by the Center for Registry and Research in Congenital Anomalies (CRIAC) at the Hospital Civil de Guadalajara “Juan I. Menchaca.” Eligibility criteria included a gestational age greater than 20 weeks, a birth weight greater than 500 g, and no evident malformations on physical examination. No reported diseases, history of cancer, or history of malformations were asked to the parents. In addition, parents were required to report Mexican ethnicity. Of the controls, 53% (n = 158) were males and 47% (n = 143) were females.

All participants’ parents or legal guardians provided informed consent. The study was approved by the Research Ethics Committee of the Hospital Civil de Guadalajara “Juan I. Menchaca” (registration number 0658/23 HCJIM2023).

**Genotype Analysis**

Genomic DNA was extracted from peripheral blood samples by the silica mini spin-column method using the QIAamp^®^ DNA Blood Mini Kit (Qiagen, Hilden, Germany). Variants in cases and controls were genotyped by TaqMan allelic discrimination assay (Applied Biosystems, Foster City, CA, USA) for the *MTHFR* C677T (SNP ID: rs1801133; Assay ID: C_1202883_20) and *MTHFR* A1298C (SNP ID: rs1801131; Assay ID: C_850486_20) polymorphisms. PCRs were analyzed on an ABI 7900HT Real-Time PCR Instrument (Applied Biosystems, Foster City, CA, USA) and performed in MicroAmp™ Fast Optical 96-Well Reaction Plates. Genotype calls were analyzed using TaqMan Genotyper Software version 1.4.0. Genotypes were assigned using the TaqMan Genotyper Autocall Algorithm (confidence level > 0.95) based on cluster separation, using an angular difference between each cluster exceeding 30°, ensuring clear cluster resolution to prevent misclassification. All genotypes were validated against no-template controls and internal controls to minimize PCR-related technical errors.

**Statistical Analysis**

The chi-square (χ^2^) test was used to compare allele frequencies and assess Hardy–Weinberg equilibrium (HWE) in the case and control groups. Associations between RB and the *MTHFR* C677T and A1298C genotypes were evaluated using Fisher’s exact test, with odds ratios (ORs) and 95% confidence intervals (CIs) calculated to estimate the strength of these associations. A recessive genetic model was selected a priori based on the functional effects previously described for the C677T polymorphism, in which homozygosity for the T allele has been associated with up to a 70% reduction in MTHFR enzymatic activity. The same analytical framework was applied to A1298C to maintain consistency between the two evaluated polymorphisms. Statistical analyses were performed with IBM^®^ SPSS statistics v27.0 (IBM Corp., Armonk, NY, USA) and R software version 4.4.1 (R Foundation for Statistical Computing, Vienna, Austria). The significance level was set at *p* < 0.05.

## 3. Results

Two variants for the *MTHFR* gene, C677T and A1298C, were studied in a group of Mexican children with RB and a group of healthy individuals as the control group. The case group consisted of 58 patients with retinoblastoma (29/50.0% male and 29/50.0% female) with a median age at diagnosis of 12 months. Unilateral retinoblastoma was present in 32 patients (55.2%), while 26 presented with a bilateral phenotype (44.8%). The primary presenting clinical signs were leukocoria in 74.1% (n = 43) and strabismus in 17.2% (n = 10) of cases, while the remaining 8.7% (n = 5) presented with other ocular signs and symptoms (including red eye, orbital inflammation, or decreased visual acuity). According to the International Classification of Retinoblastoma (ICRB), advanced intraocular tumors predominated, with 12.1% classified as Group D and 53.4% as Group E. A positive family history was documented in 1 patient (1.7%; familial bilateral retinoblastoma). Targeted germline *RB1* mutational analysis identified pathogenic germline variants in 24 patients (22 bilateral and 2 unilateral), whereas 29 patients tested negative for germline variants (all unilateral) and 5 patients were not investigated. The control group consisted of 301 healthy live-born infants enrolled at birth (age 0 days) by the Center for Registry and Research in Congenital Anomalies (CRIAC) at the Hospital Civil de Guadalajara ‘Dr. Juan I. Menchaca’. Eligibility criteria included a gestational age greater than 20 weeks, birth weight exceeding 500 g, no physical malformations upon examination, unrelated Mexican-Mestizo individuals of self-reported Mexican ethnicity by their parents/legal guardians, and no reported diseases or family history of cancer or congenital anomalies.

The distribution of genotypes and alleles in the study groups is presented in Table 1. The distribution for the genotypes C677T (χ^2^: *p* = 0.0011) and A1298C (χ^2^: *p* = 0.1358) was not in HWE for the C677T variant but it was in equilibrium for the A1298C variant for the group of cases. The distribution for the genotypes C677T (χ^2^: *p* = 0.1211) and A1298C (χ^2^: *p* = 0.7877) was in HWE for the C677T and the A1298C variant for the group of controls. Statistical analysis revealed a significant association between the *MTHFR* C677T variant and RB susceptibility under the recessive model (TT vs. CC + CT). To resolve sparse-data bias resulting from the single TT case, Firth’s penalized logistic regression was performed, confirming a significantly reduced odds of RB in TT homozygotes (OR = 0.066, 95% CI: 0.007–0.250, *p* < 0.001). This association remained statistically significant after applying a Bonferroni correction for testing two SNPs (alpha = 0.025). Similarly, the 677T allele frequency was significantly lower in cases (33.6%) than in controls (51%) (OR = 0.486, 95% CI: 0.320–0.738, *p* = 0.007). No association was found between the A1298C variant and RB under the recessive model (CC vs. AA + AC).

All samples met strict quality-control criteria, achieving overall genotype call rates of 97.8% for *MTHFR* C677T and 97.9% for A1298C. Blinding was maintained throughout, and duplicate sample concordance reached 100%. Quantitative evaluation of TaqMan allelic discrimination demonstrated clear, discrete cluster separation without evidence of cluster compression or boundary ambiguity.

For the C677T variant, the mean angles were 9.20° for the VIC/VIC (CC) cluster, 49.19° for the VIC/FAM (CT) cluster, and 84.11° for the FAM/FAM (TT) cluster, yielding inter-cluster angular differences of 39.98° (CC vs. CT) and 34.92° (CT vs. TT). Similarly, for the A1298C variant, the mean angles were 87.98° for the FAM/FAM (AA) cluster, 49.59° for the VIC/FAM (AC) cluster, and 4.81° for the VIC/VIC (CC) cluster, yielding inter-cluster angular differences of 38.39° (AA vs. AC) and 44.79° (AC vs. CC).

Stratification by clinical laterality was performed to evaluate potential disease heterogeneity between unilateral (n = 32) and bilateral (n = 26) RB cases. The *MTHFR* 677TT genotype was absent among bilateral cases and detected in 1 unilateral case, showing no statistically significant difference between subgroups (χ^2^, *p* = 0.544). Likewise, the 677T allele frequency did not differ significantly between unilateral and bilateral presentation (χ^2^, *p* = 0.745).

## 4. Discussion

The objective of the study was to investigate the association between the common variants C677T and A1298C of the *MTHFR* gene and RB in Mexican children. To the best of our knowledge, this study represents the first research investigating this association in the Mexican population.

Our results showed no association between the A1298C variant of the *MTHFR* gene and RB. Similarly, previous studies in Iranian populations [12,13] and in Brazilian populations [11] also found no association between RB and the A1298C variant of the *MTHFR* gene.

**Figure 1:**
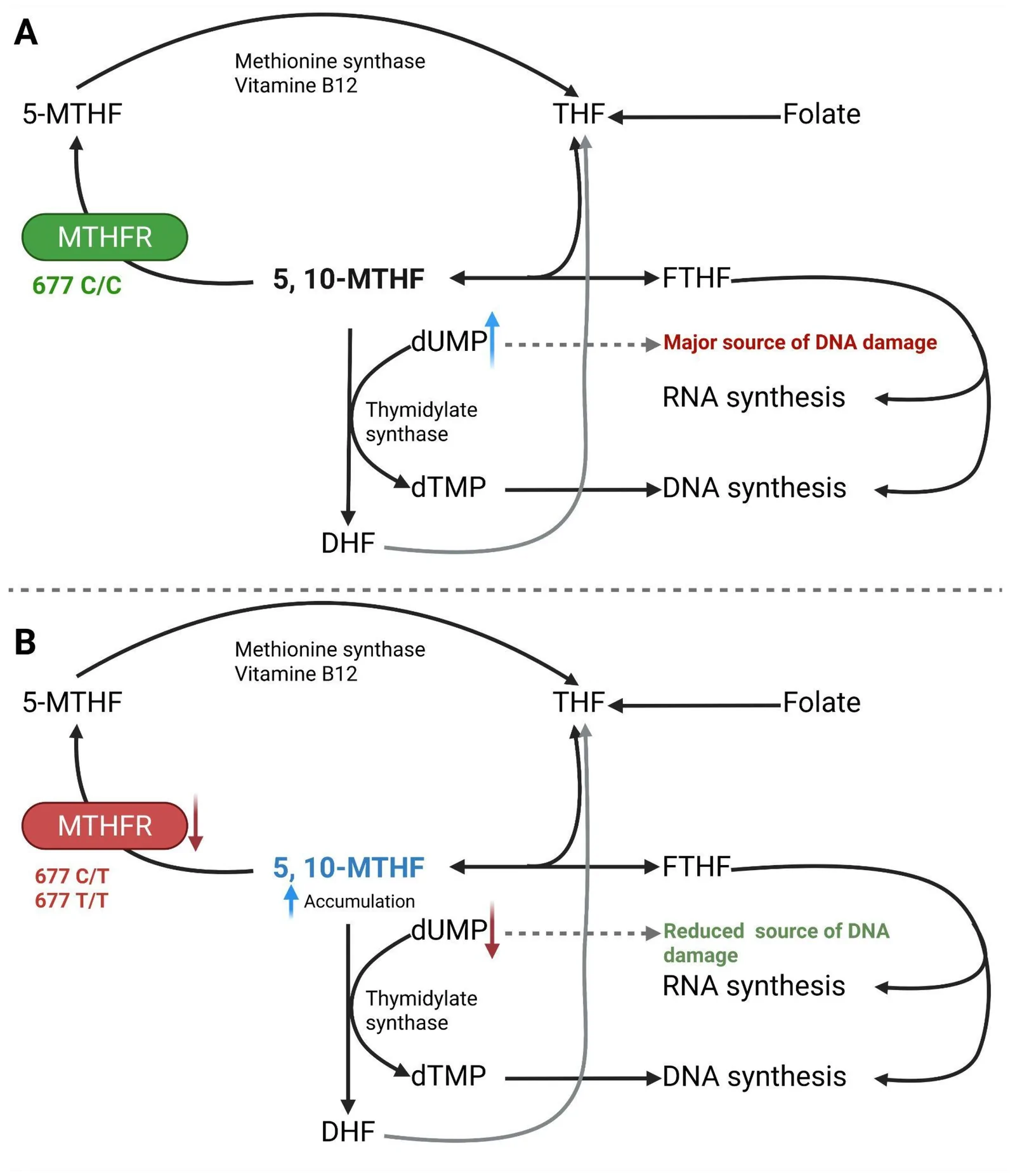
Hypothetical model of the proposed biological mechanism linking *MTHFR* variants and folate metabolism to RB susceptibility. (**A**) Schematic representation of normal MTHFR activity (677 C/C genotype). Under these conditions, reduced availability of 5,10-MTHF for the thymidylate synthase reaction favors accumulation of dUMP, increasing the likelihood of uracil misincorporation into DNA and subsequent genomic instability. (**B**) Representation of reduced MTHFR activity associated with the C677T polymorphism (677 C/T or 677 T/T genotypes). Accumulation of 5,10-MTHF enhances thymidylate synthase-mediated conversion of dUMP to dTMP, thereby reducing dUMP accumulation, limiting uracil misincorporation into DNA, and potentially exerting a protective effect against DNA damage.

Our research showed an association between RB and the C677T variant of the *MTHFR* gene under the recessive model (TT vs. CC + CT). The *MTHFR* 677TT genotype was significantly less frequent in the case group than in the control group (*p* < 0.001). Individuals who are homozygous for the T allele of the C677T variant of the *MTHFR* gene have a reduced odds (OR = 0.066, 95% CI: 0.007–0.250) of developing RB, this association remained statistically significant even after applying a Bonferroni correction for multiple testing across the two evaluated SNPs (alpha = 0.025). Similarly, the *MTHFR* 677T allele was significantly less frequent in the case group than in the control group (*p* = 0.007). This, in addition to the very low frequency of the T allele in the case group, suggests a possible protective effect of the T allele against the risk of developing RB, (Figure 1).

These results are fully consistent with meta-analytic evidence, such as the pooled analysis by Gohari et al. [14], which demonstrated a robust protective effect for C677T across diverse populations (pooled OR = 0.076, 95% CI: 0.046–0.128). While minor variations in magnitude exist across individual cohorts, several factors explain these dynamics. First, the baseline frequency of the 677T allele is exceptionally high in the Mexican population due to predominant Native American ancestry [15]. Differences in background linkage disequilibrium and genetic modifier networks may thus modulate the phenotypic expressivity of the variant across populations. Second, environmental factors, particularly dietary folate intake and mandatory national folic acid fortification, interact with reduced MTHFR activity [16,17]. While our initial univariate analysis presented a sparse-data scenario, the implementation of Firth’s penalized regression effectively resolved estimation bias, confirming a robust protective effect (*p* < 0.001) that warrants further validation in larger prospective pediatric cohorts.

Clinical heterogeneity represents an important consideration in RB, as bilateral presentation typically indicates underlying germline *RB1* pathogenic alterations [18]. Low-penetrance modifier genes in the folate metabolic pathway, such as *MTHFR*, could theoretically exert differential effects depending on whether initiation is driven by constitutional or somatic *RB1* inactivation. In our cohort, subgroup stratification by laterality revealed no significant differences in C677T variant distribution between unilateral and bilateral cases. However, because only a single case harbored the TT genotype, further multivariable modeling within clinical subgroups was constrained by statistical power. Future multi-center studies integrating precise *RB1* mutational profiling are needed to explore potential epistatic interactions between primary driver mutations and folate pathway modifiers.

Regarding the possible protective association conferred by the 677TT genotype of the *MTHFR* gene, it is known that the presence of the T allele significantly reduces MTHFR enzyme activity, and that the T allele in homozygosity can reduce MTHFR activity by up to 70% [16,17]. We hypothesize a plausible biological mechanism whereby the *MTHFR* 677T variant could modulate RB risk through reduced enzymatic activity. Specifically, lower MTHFR activity limits the reduction of 5,10-methylentetrahydrofolate (THF) to 5-methyl-THF, leading to the accumulation of 5,10-methylene-THF [19]. 5,10-methylene-THF is then used as a carbon atom donor to deoxyuridine monophosphate (dUMP) for its conversion to deoxythymidine monophosphate (dTMP) in a reaction mediated by the enzyme thymidylate synthase (TYMS) [20]. Therefore, the possible protective association of the T allele in individuals with the 677TT genotype may be due to the decrease in MTHFR enzyme activity and the consequent accumulation of 5,10-methylene-THF. This, in turn, could lead to increased conversion of dUMP to dTMP, which could be accompanied by less accumulation of dUMP, which is known to be a major source of damage to the genome when mistakenly incorporated into DNA [21]. Therefore, the cumulative reduction in dUMP may lead to decreased DNA damage and increased protection against malignancy (Figure 1) [13]. However, because serum/cellular folate levels, homocysteine concentrations, MTHFR enzymatic kinetics, DNA methylation status, and DNA damage markers were not measured in this cohort, this mechanistic model remains strictly theoretical and serves as a framework for future functional studies.

Study limitations warrant consideration. First, the small case sample (n = 58) created a sparse-data situation under recessive genetic models (a single 677TT homozygote and zero 1298CC cases), which prevented multivariable regression adjustments for age or sex without overfitting; nevertheless, parameter estimation bias and complete separation were resolved using Firth’s penalized logistic regression. Second, although 45% of cases presented with bilateral disease (indicative of germline *RB1* alterations), C677T genotype frequencies did not differ significantly by clinical laterality (*p* = 0.544), and statistical subgroup stratification by specific clinical parameters or germline *RB1* status was constrained by sample size within individual subcategories. Third, while HWE departure was restricted strictly to cases frequently reflectings true disease-allele selection, subtle population stratification or unmeasured confounders cannot be completely ruled out.

## 5. Conclusions

In summary, this case–control study conducted in a Mexican population identified an association between the *MTHFR* C677T variant and the risk of RB. Our findings suggest that the T allele may exert a possible protective association, with individuals carrying the TT genotype showing a reduced risk of developing RB. In contrast, no significant association was observed between the *MTHFR* A1298C variant and RB risk. Based on the proposed allele-specific protective mechanism, alterations in folate-dependent DNA metabolism and genomic stability warrant further investigation in RB and potentially in other cancer types.

## Figures and Tables

**Table 1:** Allele frequencies and genotype of the *MTHFR* C677T and A1298C variants in the case and control groups.

Polymorphism	Allele/Genotype	Cases (n = 58)	Controls (n = 301)	*p* Value	OR (CI)
*MTHFR* C677T	C	77 (66.4%)	295 (49%)	0.007	0.486 (0.320–0.738)
	T	39 (33.6%)	307 (51%)		
	CC	20 (34.5%)	79 (26.2%)	<0.001 *	0.066 (0.007–0.250)
	CT	37 (63.8%)	137 (45.5%)		
	TT	1 (1.7%)	85 (28.8%)		
*MTHFR* A1298C	A	97 (83.6%)	494 (82%)	0.661	0.887 (0.520–1.513)
	C	19 (16.4%)	109 (18%)		
	AA	39 (67.2%)	202 (67.1%)	0.262 *	0.263 (0.002–2.123)
	AC	19 (32.8%)	90 (29.9%)		
	CC	0 (0%)	9 (3%)		

* Odds Ratios (ORs), 95% Profile Penalized Confidence Intervals (CIs), and *p*-values for genotypes with sparse data or zero-count cells (*MTHFR* 677TT and A1298CC) were estimated using Firth’s penalized logistic regression.

## Data Availability

The data generated in the present study may be requested from the corresponding author.

## Abbreviations

The following abbreviations are used in this manuscript:

dUMP	Deoxyuridine Monophosphate
dTMP	Deoxythymidine Monophosphate
MTHFR	Methylenetetrahydrofolate Reductase
RB	Retinoblastoma
